# Investigating the potential role of metabolic resistance genes in conferring cross-resistance to pyrethroids and polycyclic aromatic hydrocarbon pollutants in the major malaria vector *Anopheles coluzzii*

**DOI:** 10.1186/s12864-025-12229-x

**Published:** 2025-11-07

**Authors:** Abdullahi Muhammad, Sulaiman S. Ibrahim, Hanafy M. Ismail, Helen Irving, Mark J.I. Paine, Charles S. Wondji

**Affiliations:** 1 Vector Biology Department, Liverpool School of Tropical Medicine, Pembroke Place, Liverpool , L3 5QA United Kingdom; 2 Centre for Biotechnology Research Bayero University, Kano, PMB 3011 Nigeria; 3 Department of Biochemistry, Bayero University, Kano, PMB 3011 Nigeria; 4 LSTM Research Unit, Centre for Research in Infectious Diseases (CRID), Yaoundé, Cameroon

**Keywords:** Cytochrome P450, Microsome, *Anopheles coluzzii*, Polycyclic aromatic hydrocarbons, Cross-resistance, Insecticide, Malaria

## Abstract

**Background:**

Polycyclic aromatic hydrocarbons (PAHs) are a class of ubiquitous and recalcitrant environmental pollutants generated from petroleum activities and/or biological conversion of organic materials. Environmental exposure of mosquitoes to these pollutants can potentially select resistance to insecticides used in public health for vector control. To understand the cross-resistance potentials between PAHs and pyrethroid insecticides, microsomal fractions prepared from *Anopheles coluzzii* mosquitoes obtained from agricultural sites and a laboratory susceptible strain, Ngousso, were tested with three major PAHs - fluorene, fluoranthene and naphthalene. Recombinant P450s previously associated with pyrethroid resistance in *Anopheles gambiae* (*CYPs 6M2*,* 6Z2*,* 6Z3*,* 9J5*,* 6P3*,* 6P4*,* 6P5*,* CYP9K1)* and *Anopheles funestus CYP6P9a* were also used to investigate metabolism of the above PAHs alongside the microsome.

**Results:**

Microsomes prepared from pyrethroid-resistant *Anopheles coluzzii* significantly *(P* = 0.001, *r =* 0.99) depleted fluorene and fluoranthene with percentage depletions of 73%±0.5 and 43%0.0 ± 2.2, respectively. A Steady-state kinetic study demonstrated that the microsome has a high affinity for fluorene with a Km and turnover of 58.69 µM ± 20.47​ and 37.016 min-^1^ ± 3.67, respectively. On the other hand, significant metabolism of fluorene up to 47.9%±2.3 (*P* = 0.001, *r* = 0.99) and 52.8%±0.8 (*P* = 0.001, *r* = 0.97) depletions were observed with recombinant *CYP6P3* and *CYP6Z3*, respectively. Other P450s showed little to no metabolism with fluorene. *CYP6Z2* and *CYP6Z3* metabolised fluoranthene with percentage depletions of 50.4%±4.9 (*P* = 0.003, *r* = 0.96) and 60.3% ±5.3 (*P* = 0.002, *r* = 0.84), respectively. However, no metabolism of naphthalene was observed with all the recombinant P450s used in this study.

**Conclusion:**

This study demonstrates that P450 monooxygenases from the malaria vectors can metabolise PAHs, highlighting the potential of these environmental pollutants selecting the P450s, driving insecticide resistance in field populations of major malaria vectors.

## Background

Insecticide resistance poses a significant threat to the gains made in malaria control through vector control tools [1–3]. Insecticide-based interventions such as the insecticide-treated nets (ITNs) and indoor residual spraying (IRS) are being challenged by the evolution/escalation of resistance even to newer chemistries [4]. Resistance to at least one insecticide has been reported in virtually all the WHO African regions, with strong indications of cross-resistance to other insecticides in most areas [2, 5, 6]. *An. coluzzii*,* An. gambiae* and *An. funestus* are the major malaria vectors in Nigeria [7], with *An. coluzzii* becoming more predominant in all climates of West/Central Africa [8]. Resistance to all classes of insecticides has been reported in various studies with near fixed frequencies of *kdr* mutations in some cases [9]. *An. coluzzii* is highly adaptive to breeding in urban areas compared to other vectors. This may be attributed to its increased resistance to osmotic stress from anthropogenic pollutants like the PAHs [10]. This suggests its widespread exposure to urban pollution [11].

When a mosquito population becomes resistant to insecticides, the same mechanism can confer resistance to other insecticides [12]. Cross-resistance is most pronounced through metabolic resistance mechanisms due to the substrate specificity and/or promiscuity [13] of the enzymes involved in the sequestration, metabolism and excretion of xenobiotics, which can lead to the metabolism of a wide range of compounds [14, 15]. The key enzymes involved in insecticide metabolism and resistance are the cytochrome P450 monooxygenases (P450s), glutathione S-transferases (GSTs) and carboxylesterases [16].

On the other hand, prior exposure to environmental pollutants and/or agrichemicals has been shown to increase selection pressure on mosquito vectors, leading to elevated levels of metabolic resistance genes, which are linked to cross-resistance to vector control insecticides. Several studies have demonstrated that survival of mosquitoes in polluted breeding sites has led to an increase in resistance and urban malaria transmission [17–19]. Members of the *Anopheles gambiae* complex are often implicated in this adaptation as they breed in polluted waters [20–22]. Hence, it is crucial to understand the molecular mechanisms through which environmental pollutants contribute to the selection of insecticide resistance in malaria vectors, thereby unravelling the cross-resistance potentials of individual compounds [23–26]. Although many studies have documented a potential link between exposure to pollutants such as polycyclic aromatic hydrocarbons (PAHs) and insecticide resistance, little is known of the underlying molecular mechanisms driving cross-resistance in *Anopheles* mosquitoes.

Microsomes are subcellular fractions generated from the ultracentrifugation of homogenised tissues containing the membrane-bound enzymes, including the cytochrome P450 monooxygenases [27]. Microsomal fractions from higher organisms have been proven very useful in the studies of drug and other compounds metabolisms, as well as toxicity studies in drug discovery and related disciplines [28–30]. Thus, they can potentially be explored in the study of insecticide cross-resistance in malaria vectors [29]. Microsomes isolated from field-resistant populations of malaria vectors can be a useful tool in determining the potential cross-resistance of newer chemistries (e.g., synthetic insecticides) and other environmental pollutants such as PAHs [31–33].

Several studies have implicated overexpression and overactivity of key *Anopheles* cytochrome P450 monooxygenases in insecticide resistance [34]. Some of these P450s have been functionally validated in vitro using heterologous expression in *E. coli* [14, 35, 36] and in vivo, using transgenic expression in Drosophila flies [37]. For example, in *An. funestus*, for example, the duplicated *CYP6P9a/b* genes are the major drivers of pyrethroid resistance and demonstrate the ability to metabolize and confer resistance to non-pyrethroid insecticides [13, 38–40]. In addition, other P450s such as *CYP9K1* [41] have been shown to metabolize a type II pyrethroid, deltamethrin, but not a structurally similar type I pyrethroid, permethrin. Others include the highly polymorphic *CYP6M7*, which was shown to metabolise pyrethroids [42] and *CYP6AA1*, which was shown to metabolise pyrethroids as well as the carbamate bendiocarb [43].

In *An. gambiae*, several P450s have also been implicated in the metabolism of pyrethroid insecticides, these include *CYP6M2*, a strong metaboliser of pyrethroids including permethrin and deltamethrin [44], *CYP6P3*, found to significantly metabolize types I and II pyrethroids [45], *CYP6P5* located on the pyrethroid resistance locus with appreciable copy number variations [46] in the CYP6 cluster [47]. Other important pyrethroid-associated P450s include *CYP6Z3* [48] and *CYP6Z2*, which are found to have a broad range of substrate specificity, suggesting potential roles in survival at the larval stage [49]. *CYP9K1* [50] and *CYP9J5* [51] are also involved in conferring resistance to pyrethroids.

Sixteen PAHs have been reported as being of environmental concern by the US Environmental Protection Agency due to their persistence and toxicity. The smaller PAHs, with fewer benzene rings and no functional groups substituting the hydrogens in their structures, tend to be more persistent in the environment due to their poor microbial bioremediation [52, 53]. These PAHs also have greater access to the cellular membrane of mosquito larvae in the breeding sites due to their higher hydrophobicity, an important requisite for easy access to the lipid bilayer membrane. The main aim of the current study is to investigate the cross-resistance potentials between this class of ubiquitous pollutants and public health insecticides using recombinant P450s and microsomes isolated from resistant and susceptible strains of *An. coluzzii*. The study informs on the role of these compounds as drivers of metabolic insecticide resistance in *An. coluzzii.* To do that, three lower molecular weight PAHs were selected for these experiments due to their characteristics. Naphthalene with two fused benzene rings, fluorene with two benzene rings fused to a five-membered ring and fluoranthene, consisting of a naphthalene moiety and a benzene ring connected by a five-membered ring.

To study the cross-resistance between PAHs and pyrethroids, a P450 panel comprising *An. gambiae CYPs 6Z2*,* 6Z3*,* 6P5*,* 9K1*,* 6M2*,* 9J5*,* 6P3 and 6P4*, and *An. funestus CYP6P9a* was investigated for metabolic activities toward the PAHs and pyrethroid insecticides.

Isolated microsomes prepared from the field population as well as from the fully susceptible *An. coluzzii* lab colony, Ngousso were used in metabolism assays to investigate cross-resistance between pyrethroid insecticides and the three PAHs. In addition, the above P450s were heterologously expressed in *E. coli* and investigated for their potential to confer cross-resistance to these two unrelated groups of compounds.

## Methods

### Mosquito collection and rearing

Blood-fed, indoor resting female *Anopheles* mosquitoes were caught using electric aspirators (John W. Hock, Florida, USA) in Auyo town, Auyo Local Government, Jigawa State, Nigeria in September 2019. This is a highly irrigated site whose details have been described earlier [9]. The F_0_ female mosquitoes were morphologically identified [54] as members of *Anopheles gambiae* complex and kept in standard insectary conditions of 25 °C and 75% relative humidity and 12:12 h light: dark cycles, and maintained on 10% sucrose solution until they were gravid. Gravid females were individually forced to lay eggs [55] and the eggs were transported to Liverpool School of Tropical Medicine for the downstream analysis. Using the genomic DNA extracted from the F_0_ individuals, species were confirmed using SINE 200 PCR [56]. Eggs were pooled into trays and allowed to hatch. The Auyo population is highly resistant to pyrethroids and therefore used for this study to understand the cross-resistance potentials between pyrethroids and PAHs. The laboratory susceptible *Anopheles coluzzii* colony, Ngousso [57], which is fully susceptible to all insecticides, was used for comparison to the field resistant populations.

### Microsome preparation

The two strains of *Anopheles coluzzii* Auyo and Ngousso were reared in the insectary under standard insectary conditions to generate a substantial number of fourth instar larvae for the microsome preparations. Microsome preparation was conducted according to the method reported earlier with some modifications [58]. About 600 of 4th instar larvae were homogenised in 20 ml of ice-cold potassium phosphate buffer, pH 7.4, that has been supplemented with 1x protease inhibitor (Roche complete, ultra EDTA free, Sigma Aldrich, MA, USA). The larvae were homogenized in a 40 ml Dounce homogenizer equipped with a loose B pestle (Wheaton Science, Millville, NJ, USA). Filtration was conducted with layers of nylon filters to remove the residual debris. The filtrate was initially centrifuged at 10,000 xg for 10 min at 4 ˚C to separate the cytosolic fractions (supernatant) from other heavier cellular components. The supernatant was then centrifuged at 200,000 xg for 45 min at 4 ˚C to collect the pellet and discard the new supernatant. The pellet was reconstituted in 0.1 M potassium phosphate buffer, pH 7.4, containing 20% glycerol. Total protein content of the microsomes was determined using the Bradford method [59]. Cytochrome P450 content (Cary WinUV Software, Agilent Technologies) and microsomal P450 activity were also determined using spectral activity assay [60, 61].

### Heterologous expression of recombinant P450s

The recombinant P450s used in this study were acquired from the enzyme characterization group (ECG) leader, Dr. Mark J.I. Paine of the Vector Biology Department, Liverpool School of Tropical Medicine. All the P450s were expressed from field-resistant populations of *An. gambiae* except for CYP6P9a, which was expressed from *An. funestus.* The heterologous expression of the P450s was conducted according to the previously described approaches [35, 44, 49]. Briefly, P450s were expressed by using *ompA* and *pelB* signal sequences to direct P450 and *An. gambiae* CPR (AgCPR), respectively, to the inner membrane of *E. coli* a functional monooxygenase. The P450 sequences were amplified from cDNA and fused to the ompA signal peptides in simple PCR reactions. This is followed by ligation of the digested fragments into linearized expression vector pCWori+, thereby creating a construct pB13:*ompA + 2*-P450. Competent JM109 cells were co-transformed with plasmids pB13:*ompA + 2*-P450 and pACYC-AgCPR for the expression of functional P450 and *An. gambiae* CPR, respectively. Colonies carrying the two plasmids as confirmed by the colony PCR are used for a 12–14 h starter culture overnight. Typically, 0.2 L cultures were supplemented with 2 mL from the starter cultures and incubated at 37 °C and 200 rpm shaking while the absorbance at 600 nm was being monitored. When the OD reached 0.6–0.8, the cultures were cooled down to 25 °C for 30 min with shaking at 150 rpm. Induction with 1 mM isopropyl β-D-1-thiogalactopyranoside (IPTG) and 0.5 mM 5-aminolaevulinic acid (ALA) was conducted. Determinations of the P450 content and CPR activity were conducted using the procedure of [61] and [60], respectively.

### Measurement of microsomal P450 activity using model probe substrate

P450 activity was determined in the prepared microsomes from both Auyo and Ngousso strains using diethoxyfluorescein (DEF) as the model fluorogenic substrate using the methods earlier described [15]. Briefly, the enzyme-buffer mix consisted of 0.05 µM of cytochrome P450 (microsomes), 50 mM potassium phosphate buffer, pH 7.4, 0.5 µM cytochrome b5 and 10 µM DEF. To initiate the reactions in a 96-well plates (Thermo Labsystems, Basingstoke, UK), a NADPH (nicotinamide adenine dinucleotide) regeneration mix (consisting of 1mM glucose-6-phosphate, 0.1 M NADP+, 0.25mM magnesium chloride, IU/ml glucose-6-phosphate dehydrogenase and 50 mM potassium phosphate buffer pH 7.4) was added in the positive replicates whereas the same buffer with no NADPH was added in the negative wells. Using the excitation (482 nm) and emission (520 nm) wavelengths of DEF, the absorbance was read for 15 min, and the relative fluorescence unit per min per picomole of the P450 (RFU/min/pmol of P450) was determined.

### In vitro metabolism assay of PAHs with heterologously expressed P450s

To compare the metabolic activity of the microsomes and the recombinant cytochrome P450s towards PAH metabolism assays were conducted side by side as described earlier [38, 43]. The NADPH regeneration mixture (consisting of 0.1 M NADP+, 50mM potassium phosphate buffer, pH 7.4, 1 mM glucose-6-phosphate, 0.25 mM magnesium chloride and 1U/ml glucose-6-phosphate dehydrogenase) was used in the positive replicates. For the negatives, the same buffer with no added NADP + was used. Enzyme-buffer mix comprised of 0.05 µM recombinant P450 or microsome, 0.4 µM cytochrome b5, 50 mM potassium phosphate buffer pH 7.4 and 20 µM of the PAH substrates. To initiate the reactions, regeneration mixture was added to the enzyme-buffer mix in a 1:1 ratio (total of 200 µl) following activation for 5 min at 30 °C and 1200 rpm shaking. The reaction was run for 2 h at 30 °C with 200 rpm shaking. To stop the reaction, 200 µl of ice-cold High Performance Liquid Chromatography (HPLC) grade acetonitrile was added, followed by additional shaking for 5 min to precipitate the proteins. The mixture was kept on ice for 10 min before centrifuging at 16,000xg for 20 min. The supernatants were filtered through 0.45 μm PTFE filters (ThermoFisher Scientific, MA, USA) and filtrate transferred to HPLC vials for onward analysis.

Using the Agilent HPLC 1200 infinity series, analysis of the PAHs metabolism was conducted. The HPLC conditions consisted of mobile phases of HPLC grade acetonitrile and water in the ratio of 80/20 and detected at a wavelength of 254 nm. 50 µl of the supernatant was injected on the HPLC and peaks were separated on a 250 × 4.6 mm (5 μm) Supelcosil LC-18-DB column (Supelco, Sigma-Aldrich, Gillingham, U.K.) in a 20 min run. Percentage depletions for each compound were calculated in both the microsomal and recombinant P450 studies. Using pair-wise t-test, comparisons were made between the positives (+ NADPH) (where reactions were expected) and the negative controls (-NADPH) (where no reactions were expected due to the absence of NADPH source in the mix). The effect size between the two means of each comparable group was calculated [62]. Comparisons were also made using the same parameters between depletions in field resistant and laboratory susceptible microsomal metabolism of PAHs.

### Fluorene turnover assay using microsomes

Because of the higher percentage of depletion observed in the Auyo microsomal metabolism of fluorene, it was chosen to be analysed further for its turnover and kinetic studies. To understand the turnover rates of the fluorene depletions, reaction run time was varied between 15 and 150 min. Specific points used were 15, 30, 45, 60, 90, 120 and 150 min while the fluorene concentration was maintained at 30 µM in all these time points.

### Determination of steady-state kinetics parameters for the microsomal metabolism of fluorene

For the determination of steady-state kinetic parameters, assays were conducted for 30 min with 0.05 µM microsomal P450 while varying the concentrations of fluorene (0–600 µM). Kinetics plot of velocity against the substrate concentrations was made using the least squares non-linear regression in GraphPad Prism 6.03 Software (GraphPad Inc., La Jolla, CA, USA) that fits into the canonical Michaelis-Menten model as previously described for pyrethroid insecticides [43].

## Results

### P450 content and activity in microsomal fractions

**Fig. 1:**
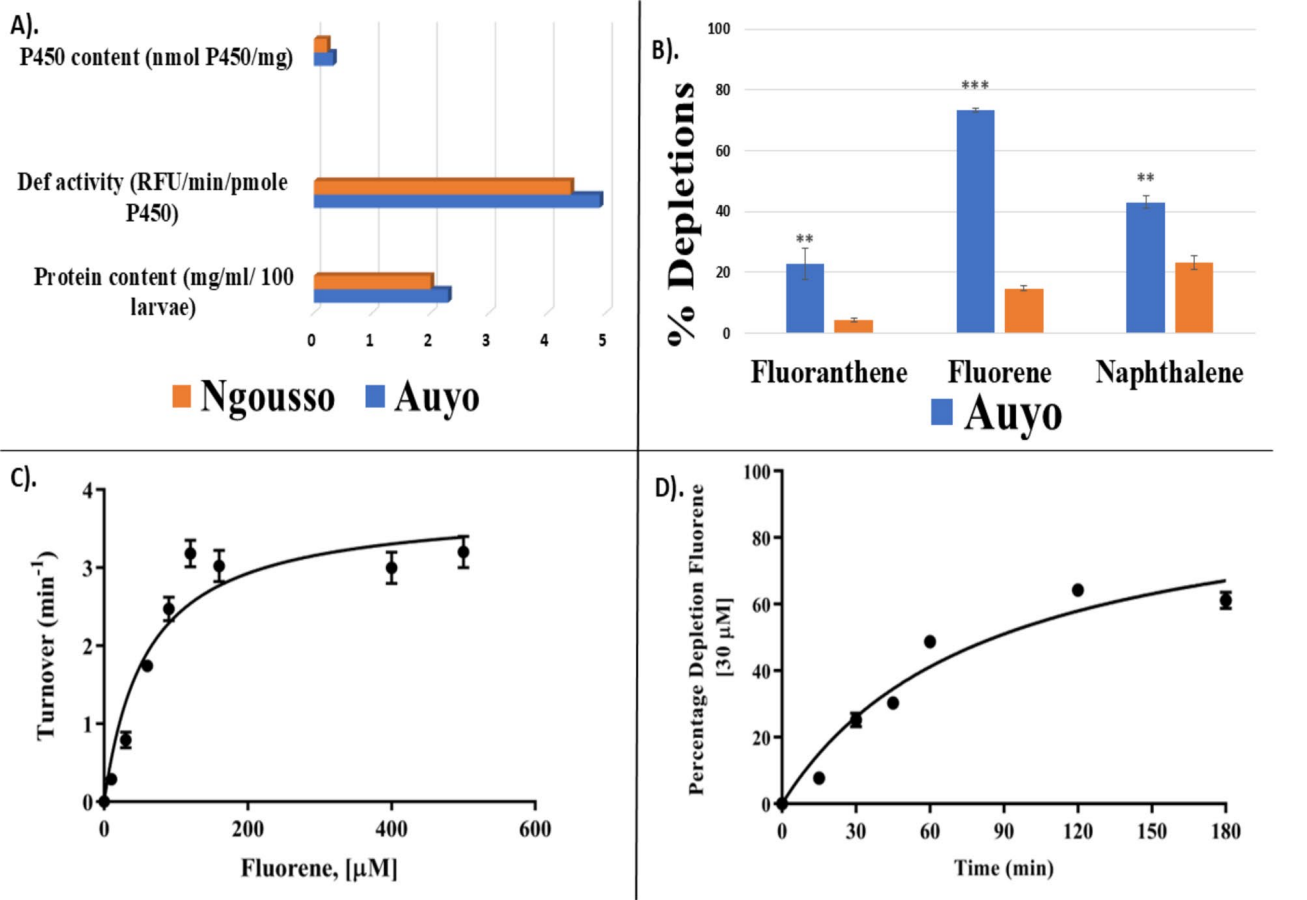
Catalytic activity of the Auyo and Ngousso microsomes towards PAHs and the enzyme kinetics studies of the Auyo microsomes with fluorene as substrate. **A** Def activity and the protein content of the isolated microsomes. **B** Percentage depletions of the metabolism of the select PAHs by the microsomes isolated from the pyrethroid-resistant and susceptible strains of *Anopheles coluzzii.* Results are presented as mean ± SD of three replicates of the positives (+ NADPH) compared to the negatives (-NADPH). ** Significant difference between the Ngousso and Auyo percentage depletions for each substrate. **C** Michaelis-Menten plot of fluorene metabolism by microsomes isolated from the field-resistant strain (Auyo). **D** Time course of fluorene turnover, substrate concentration 20 µM

Microsomal preparations from both the field-resistant strain (Auyo) and laboratory susceptible strain (Ngousso) of *An. coluzzii* produced P450 contents of 0.33 nmol P450/mg and 0.226 nmol P450/mg m, respectively. The microsomes were screened for P450 activities using the model fluorogenic substrate diethoxyfluorescein (DEF). DEF activities of 4.9 RFU/min/pmole P450 and 4.4 RFU/min/pmole P450, respectively (Fig. 1A), were established for the Auyo and Ngousso microsomes, confirming the presence of functional cytochrome P450s. A total protein content of 2.0 mg/ml/100 larvae and 2.3 mg/ml/100 larvae were also determined for the Auyo and Ngousso microsomal preparations, respectively (Fig. 1A).

### Metabolism of the PAHs by microsomal fractions

Microsomal P450 demonstrated metabolic activity towards PAHs (fluorene, fluoranthene and naphthalene) with higher percentage depletions observed in the Auyo microsomes. For example, significant depletion of fluorene (73.3 ± 0.44%, *P* = 0.0001, *r* = 0.99) was seen with the Auyo microsome compared to the Ngousso microsome (Fig. 1B). A Similar profile was obtained with fluoranthene, with 22.8 ± 5.2% significantly depleted by Auyo microsome (*P* = 0.001, *r* = 0.97), compared with the Ngousso microsome. Similar pattern was observed with naphthalene.

Steady-state kinetic parameters were investigated with fluorene, the most significantly depleted PAH by the Auyo microsomes. Turnover of 37.02 min^−1^ ± 3.67 was recorded. The microsome demonstrated moderate affinity towards fluorene (Fig. 1C), with a Km value of 58.69 µΜ ± 20.47. Catalytic rate of (K_cat_) of 4.196 min^−1^ ± 0.436 with a corresponding high catalytic efficiency of 0.0715 ± 0.026 min^−1^ µM^−1^ (Fig. 1D).

### The metabolic activity of recombinant pyrethroid-associated P450s towards PAHs

**Fig. 2:**
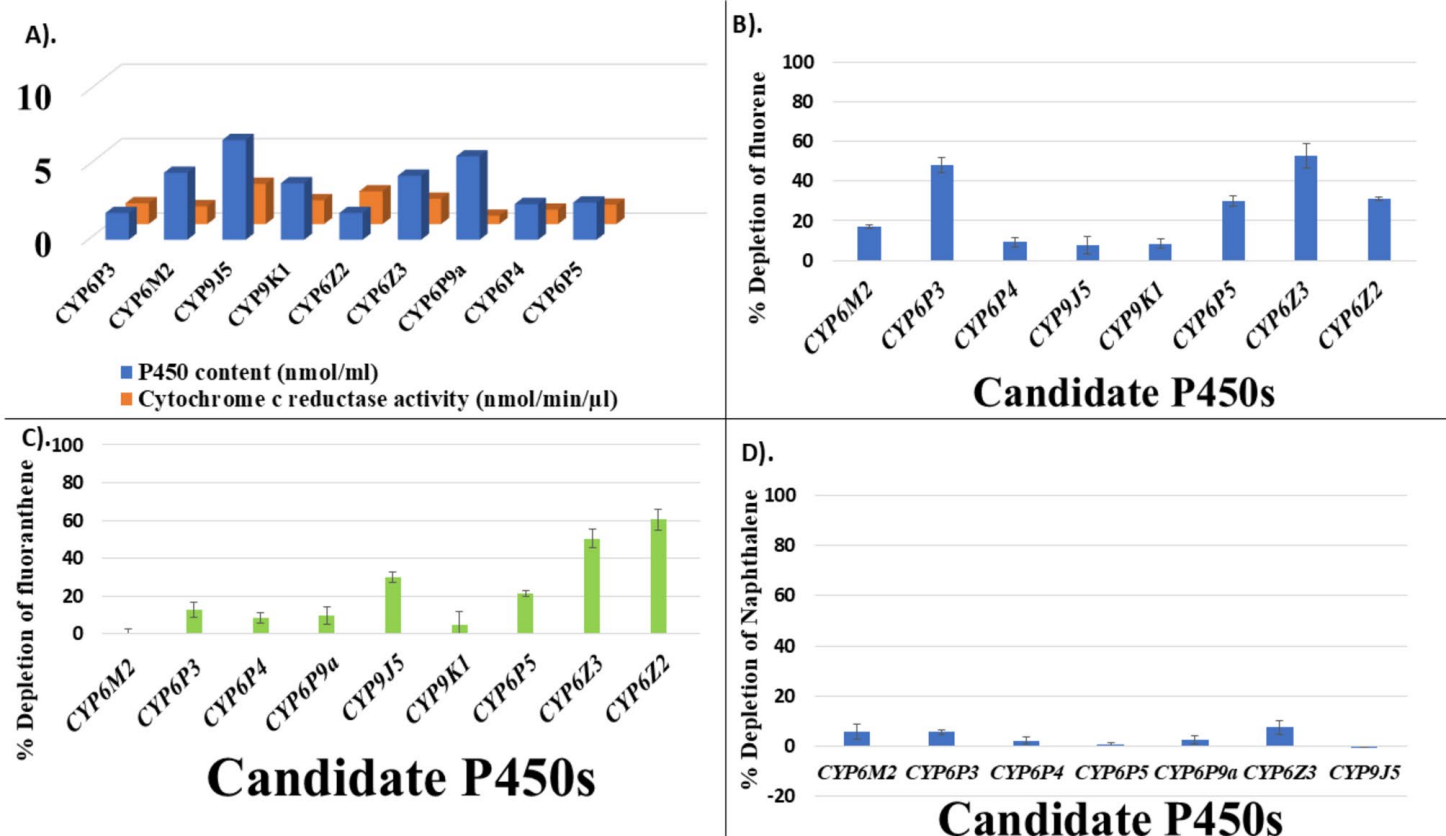
Metabolism of PAHs by pyrethroid-associated recombinant cytochrome P450 monooxygenases. Details of the P450 membranes used in the study including the P450 content and CPR activity (**A**). Percentage depletions of fluorene (**B**), fluoranthene (**C**) and naphthalene (**D**) for the various recombinant cytochrome P450s. Values are presented as mean ± S.D. of three technical replicates. Percentage depletions were calculated by comparison of positive (+ NADPH) vs. negative (-NADPH) reactions in triplicate

To understand the potential cross-resistance liabilities between the recalcitrant environmental pollutant PAHs and pyrethroids, cytochrome P450s previously implicated in pyrethroid resistance/metabolism were recombinantly expressed and used for metabolism assays with PAHs. The pyrethroid metabolism associated P450s showed varied levels of metabolism towards the three select PAHs, naphthalene (Fig. 2D), fluorene (Fig. 2B) and fluoranthene (Fig. 2C). For example, none of the P450s used in this study depleted up to 10% of naphthalene, suggesting no cross-resistance towards this PAH and pyrethroid insecticides through the P450 metabolism route (Fig. 2D). On the other hand, fluoranthene was significantly depleted by the recombinant CYP6Z2 and CYP6Z3, with percentage depletions of 60% ± 4.9 and 50.4% ± 5.3, respectively (Fig. 2C). In the case of fluorene, highest depletions were seen with the recombinant CYP6Z3 (52.8 ± 0.8%) and CYP6P3 (47.9 ± 2.3%) (Fig. 2B), with much lower depletions obtained from CYP9K1 (8.5% ± 2.5), CYP6P4 (9.3% ± 4.3), and CYP9J5 (7.7% ± 2.5).

## Discussion

Chemical insecticides used in vector control tools and agriculture are considered the major drivers of insecticide resistance alongside environmental pollutants [51, 63–65]. Microsomal fractions isolated from pyrethroid-resistant and susceptible strains of *An. coluzzii* were used to study the cross-resistance between pyrethroids and PAHs as a class of ubiquitous environmental pollutants. Microsomes are the cytoplasmic fractions of the cells containing the membrane-bound enzymes, including the cytochrome P450s, and their use in drug discovery and toxicity studies has been well documented [28, 30] compared to vector biology studies. They can thus be explored for the studies of insecticide resistance liabilities without the need for recombinant expression of individual P450s. Successful isolations of microsomes from different insect species have been reported, mainly from *Aedes aegypti* mosquitoes [32, 33, 66–68]. In the present study, microsomes with substantial P450 activity and content were successfully isolated from the larvae of pyrethroid-resistant and susceptible strains of *An. coluzzii.* The P450 yield and activity were higher (0.33 nmol P450/mg protein for the resistant and 0.226 nmol P450/mg for the susceptible) than those seen in a recent study [66] but lower than what was obtained from southern armyworm (*Spodeptera eridania)* microsomal preparations [69].

Generally, metabolism of PAHs by cytochrome P450s in different organisms, including insects, proceeds via C-H activation [70, 71]. For example, the human *CYP3A4* has been shown using HPLC coupled with NMR to generate a mono-hydroxylated product, fluorenol and a 9-oxo substituted product, fluorenone, from the fluorene parent compound [70]. The microsomal fractions from pyrethroid-resistant strains metabolised all three PAHs significantly more than the pyrethroid susceptible ones, suggesting there was pyrethroid cross-resistance potential to environmental pollutants. In a related study [33] in *Aedes aegypti*, the microsomes from pyrethroid-resistant strains metabolised more permethrin than their susceptible counterpart. These findings suggest that prior exposure to pyrethroids can lead to resistance to other pollutants/insecticides and vice-versa.

The highest percentage depletion in both studies with microsomes and recombinant P450s was observed in the Auyo microsomal metabolism of fluorene (73.3 ± 0.4%) and was thus further characterized to understand the kinetics and turnover of this reaction. The Km (58.69 ± 20.47 µM) Kcat (4.20 ± 0.44 min^−1^) were higher than those previously obtained with recombinant P450s metabolism of insecticides. This might be because microsomal fractions contain a mixture of many P450s with varied affinity towards the substrate, thereby compounding the catalytic effects. Examples include the case of metabolism of ecdysone by microsomes of the African migratory locust [72], metabolism of permethrin and deltamethrin by the duplicated *An. funestus* CYP6P9a/b [42], permethrin and deltamethrin metabolism by *An. funestus* CYP6AA1 [43] and deltamethrin metabolism by *An. minimus* CYP6AA3.

Cytochrome P450 monooxygenases, are important Phase I enzymes [73] in the metabolism of xenobiotics and have been implicated as the major players in conferring resistance to insecticides in disease vectors. A large number have been extensively characterized and found to confer resistance to certain insecticides through metabolism, sequestration and excretion of the soluble metabolites. Some of the notable ones associated with pyrethroid resistance in *An. gambiae* and *An. funestus* were investigated for their potential in metabolising PAHs, thereby understanding their cross-resistance abilities. CYP6P3 is one of the most extensively characterized P450s in *An. gambiae* [74, 75] and promiscuous in its ability to metabolise a wide range of compounds, including type I and II pyrethroids [45]. In this study, it metabolised fluorene and fluoranthene with more than 40% depletion, suggesting its potential role in cross-resistance between PAHs and pyrethroids. *An. funestus* CYP6P9a is an orthologue of the *An. gambiae* CYP6P3 and equally very promiscuous in metabolising a wide range of pyrethroid and non-pyrethroid insecticides, however, it did not metabolise the PAHs [37, 38, 40]. Furthermore, despite the major role of CYP6M2 in pyrethroid metabolism and resistance [44], there was no activity observed with all three select PAHs, suggesting no direct activity on PAHs despite its role in pyrethroid metabolism.

CYP6Z2 serves as an important marker of pyrethroid resistance although it metabolises the carboxylesterase metabolites of pyrethroid metabolism (3-phenoxybenzoic alcohol and 3-phenoxybenzaldehyde) rather than the parent pyrethroid insecticides [49, 76]. Both CYP6Z2 and CYP6Z3 demonstrated strong metabolic activity towards fluorene and fluoranthene, indicating their potential role in the survival of *An. gambiae* in polluted breeding sites. Earlier studies have suggested that the CYP6Z family of cytochrome P450s in *An. gambiae* are most notably expressed at the early life stages of larvae and pupae [76, 77]. This means they inherently help mosquitoes’ timely survival in polluted breeding sites due to their characteristic broad range substrate specificity capable of metabolizing a wide range of compounds, including plants’ secondary metabolites [78]. This similar trend was also reported in *Aedes aegypti* mosquito’s CYP6Z8, an orthologue of the *An. gambiae* CYP6Z2 showing catalytic activity on a broad range of substrates, including α-naphthoflavone, resveratrol, and diethylstilbestrol [76]. Naphthalene was not metabolized by any of the P450s used in this study, indicating the lack of cross-resistance potentials between pyrethroids and naphthalene. This may be related to its characteristic chemical structure that gives it an additional stability compared to higher molecular weight PAHs. A similar trend has also been observed in the case of some recombinant P450s metabolising some compounds but not others in the same group. For example, *An. funestus* CYP9K1 metabolises type II pyrethroids (deltamethrin) but not type I pyrethroids like permethrin [41]. Another example is also the metabolism of all pyrethroids by recombinant *An. arabiensis* CYP6P4 except deltamethrin (a type II pyrethroid. Here, in both microsome and recombinant P450s studies, fluorene was more highly metabolized than fluoranthene, while naphthalene remained consistently the least metabolized of them all. This differential metabolism may be related to the fact that naphthalene, with lower molecular weight, was less prone to metabolism by *Anopheles* P450s compared to fluorene and fluoranthene. The findings in this study indicate the potential mechanisms through which environmental pollutants can serve as additional selection pressure in the evolution/escalation of insecticide resistance. This will have an implication on the deployment of insecticide resistance management strategies. However, some of the major limitations include the failure to establish the identity of metabolites generated by P450 depletion of PAHs to rule out a potential bioactivation scenario. In *vivo* studies with PAHs to ascertain phenotypes would also have added value to the findings. Based on our findings, we recommend using synergists in vector control tools, as the P450s (and other metabolic genes) have evolved to have a wider range of substrate specificities. Another recommendation is paying attention to environmental pollutants in resistance management strategies instead of only focusing on insecticides.

## Conclusion

Cytochrome P450s associated with pyrethroids resistance were found to metabolise PAHs in metabolic assays, confirming the potential for cross-resistance between these pollutants and insecticides of public health importance. These findings highlight the importance of the early assessment of the potential cross-resistance liabilities of P450s associated with pyrethroid or other xenobiotic metabolism to new chemistries being introduced for vector control. Future studies that seek to establish the resistance/or susceptibility of malaria vectors to major environmental pollutants will be of great importance to resistance management strategies currently in use. In *vivo* validation of the role of metabolic P450s in the metabolism/resistance to pollutants is also recommended. Wider studies that include many metabolic enzymes, including the P450s, GSTs, and carboxylesterases, to give a better picture of the cross-resistance relationship between environmental pollutants and public health insecticides.

## Data Availability

Data sharing does not apply to this article as no datasets were generated or analysed during the current study. No new sequencing has been conducted in this study as such no sequences have been deposited in any repository.

## Abbreviations

ALA: 5-aminolaevulinic acid
CYP: Cytochrome P450 monooxygenase
DEF: Diethoxyfluorescein
DNA: Deoxyribonucleic acid
EDTA: Ethylenediaminetetraacetic acid
GSTs: Glutathione S-transferase
HPLC: High performance liquid chromatography
IPTG: Isopropyl β-D-1-thiogalactopyranoside
IRS: Indoor residual spraying
ITNs: Insecticide-treated nets
NADPH: Nicotinamide adenine dinucleotide phosphate
PAHs: polycyclic aromatic hydrocarbons
PCR: Polymerase chain reaction
PTFE: Polytetrafluoroethylene
SINE: Short interspersed elements
WHO: World health organization

## References

[1] Ranson H, Lissenden N. Insecticide resistance in African *Anopheles* mosquitoes: a worsening situation that needs urgent action to maintain malaria control. Trends Parasitol. 2016;32(3):187–96. 10.1016/j.pt.2015.11.010. 26826784 10.1016/j.pt.2015.11.010

[2] WHO. World Malaria Report 2021. World Malaria report Geneva: World Health Organization. (2021). Licence: CC. 2021. 2013–2015 p.

[3] Riveron JM, Tchouakui M, Mugenzi L, Menze BD, Chiang M, Wondji CS. Insecticide resistance in Malaria Vectors: an update at a global scale. In: Towards Malaria Elimination - A Leap Forward. Intech open science open mind. 2018. pp. 137–44. Available from: http://www.intechopen.com/books/trends-in-telecommunications-technologies/gps-total-electron-content-tec- prediction-at-ionosphere-layer-over-the-equatorial-region%0AInTec.

[4] Coleman M, Hemingway J, Gleave KA, Wiebe A, Gething PW, Moyes CL. Developing global maps of insecticide resistance risk to improve vector control. Malar J. 2017;16(1):1–9. 28222727 10.1186/s12936-017-1733-z PMC5320685

[5] Corbel V, Guessan RN. Distribution, mechanisms, impact and management of insecticide resistance in Malaria Vectors: a pragmatic review. In: *Anopheles* mosquitoes - New insights into malaria vectors. Intech open science open mind. 2013. pp. 579–633. Available from: http://www.intechopen.com/books/anopheles-mosquitoes-new- insights-into-malaria-vectors%0AInterested.

[6] WHO. Global report on insecticide resistance in malaria vectors: 2010–2016. 2016. 2010–2016 p.

[7] Okorie PN, Mckenzie FE, Ademowo OG, Bockarie M, Kelly- L. Nigeria *Anopheles* vector database: an overview of 100 years ’ research. PLoS ONE. 2011;6(12):6–7. 10.1371/journal.pone.0028347 PMC3230596 22162764

[8] Ibrahim SS, Fadel AN, Tchouakui M, Terence E, Wondji MJ, Tchoupo M, et al. High insecticide resistance in the major malaria vector *Anopheles coluzzii* in Chad Republic. Infect Dis Poverty. 2019;8(1):1–12. 31796068 10.1186/s40249-019-0605-x PMC6892245

[9] Ibrahim SS, Manu YA, Tukur Z, Irving H, Wondji CS. High frequency of Kdr L1014F is associated with pyrethroid resistance in *Anopheles coluzzii* in Sudan savannah of northern Nigeria. BMC Infect Dis. 2014;14(1):1–9. 25127882 10.1186/1471-2334-14-441 PMC4147187

[10] Fifer JE, Amoa-Bosompem M, Nelson D, Terner ER, Clifford AJ, Tan S, et al. Genomics of urban adaptation and exaptation in mosquitoes and consequences for vectorial capacity. Curr Opin Insect Sci. 2025;70:101384. 10.1016/j.cois.2025.101384. 40348056 10.1016/j.cois.2025.101384 PMC12337636

[11] Tene Fossog B, Ayala D, Acevedo P, Kengne P, Ngomo Abeso Mebuy I, Makanga B, et al. Habitat segregation and ecological character displacement in cryptic African malaria mosquitoes. Evol Appl. 2015;8(4):326–45. 25926878 10.1111/eva.12242 PMC4408144

[12] WHO. Test procedures for insecticide resistance monitoring in malaria vector mosquitoes second edition. 2016. 1–48 p.

[13] Tchouakui M, Ibrahim S, Mangoua K, Thiomela R, Assatse T, Ngongang-Yipmo S, et al. Substrate promiscuity of key resistance P450s confers clothianidin resistance whilst increasing chlorfenapyr potency in malaria vectors. 114566 ISSN 2024;43(8):2211–47. 10.1016/j.celrep.2024.114566. 10.1016/j.celrep.2024.114566 PMC11372441 39088320

[14] Yunta C, Hemmings K, Stevenson B, Koekemoer LL, Matambo T, Pignatelli P, et al. Cross-resistance profiles of malaria mosquito P450s associated with pyrethroid resistance against WHO insecticides. Pestic Biochem Physiol. 2019;161(June):61–7. Available from: 10.1016/j.pestbp.2019.06.007. 10.1016/j.pestbp.2019.06.007 31685198

[15] Yunta C, Grisales N, Nász S, Hemmings K, Pignatelli P, Voice M, et al. Pyriproxyfen is metabolized by P450s associated with pyrethroid resistance in *An. gambiae*. Insect Biochem Mol Biol. 2016;78:50–7. 27613592 10.1016/j.ibmb.2016.09.001 PMC6399515

[16] Liu N. Insecticide resistance in mosquitoes: impact, mechanisms, and research. Annu Rev Entomol. 2015;60:537–59. 25564745 10.1146/annurev-ento-010814-020828

[17] Azrag RS, Mohammed BH. *Anopheles arabiensis* in Sudan: a noticeable tolerance to urban polluted larval habitats associated with resistance to Temephos. Malar J. 2018;17(1):1–11. Available from: 10.1186/s12936-018-2350-1. 10.1186/s12936-018-2350-1 PMC5960190 29776357

[18] Hay SI, Ox O, Ox O, Snow RW. Urbanization, malaria transmission and disease burden in Africa Simon. Nat Rev Microbiol. 2011;3(1):81–90. 10.1038/nrmicro1069 PMC3130901 15608702

[19] Padilla JC, Chaparro PE, Molina K, Arevalo-Herrera M, Herrera S. Is there malaria transmission in urban settings in Colombia? Malar J. 2015;14(1):1–9. 26573620 10.1186/s12936-015-0956-0 PMC4647453

[20] Kamdem C, Tene Fossog B, Simard F, Etouna J, Ndo C, Kengne P, et al. Anthropogenic habitat disturbance and ecological divergence between incipient species of the malaria mosquito *Anopheles gambiae*. PLoS ONE. 2012. 10.1371/journal.pone.0039453. 22745756 10.1371/journal.pone.0039453 PMC3382172

[21] Vicente JL, Clarkson CS, Caputo B, Gomes B, Pombi M, Sousa CA, et al. Massive introgression drives species radiation at the range limit of *Anopheles Gambiae*. Sci Rep. 2017;7(June 2016):1–13. 28417969 10.1038/srep46451 PMC5394460

[22] Oliver SV, Brooke BD. The effect of metal pollution on the life history and insecticide resistance phenotype of the major malaria vector *Anopheles arabiensis* (Diptera: Culicidae). Acta Trop. 2018;188:152–60. 30179608 10.1016/j.actatropica.2018.08.030

[23] Poupardin R, Riaz MA, Jones CM, Chandor-Proust A, Reynaud S, David JP. Do pollutants affect insecticide-driven gene selection in mosquitoes? Experimental evidence from transcriptomics. Aquat Toxicol. 2012;114–115:49–57. Available from: 10.1016/j.aquatox.2012.02.001. 10.1016/j.aquatox.2012.02.001 22406618

[24] N’Dri BP, Heitz-Tokpa K, Chouaïbou M, Raso G, Koffi AJ, Coulibaly JT, et al. Use of insecticides in agriculture and the prevention of vector-borne diseases: population knowledge, attitudes, practices and beliefs in Elibou, South Côte d’Ivoire. Trop Med Infect Dis. 2020. 10.3390/tropicalmed5010036. 32121510 10.3390/tropicalmed5010036 PMC7157594

[25] Djouaka RF, Bakare AA, Bankole HS, Doannio JM, Coulibaly ON, Kossou H, et al. Does the spillage of petroleum products in *Anopheles* breeding sites have an impact on the pyrethroid resistance? Malar J. 2007;6(1):159. 10.1186/1475-2875-6-159. 18053173 10.1186/1475-2875-6-159 PMC2222605

[26] Clark BW, Di Giulio RT. *Fundulus heteroclitus* adapted to PAHs are cross-resistant to multiple insecticides. Ecotoxicology. 2012;21(2):465–74. 10.1007/s10646-011-0807-x. 22037695 10.1007/s10646-011-0807-x PMC3278525

[27] Jia L, Liu X. The conduct of drug metabolism studies considered good practice (part II): in vitro experiments. Curr Drug Metab. 2007;8(8):822–9. 18220563 10.2174/138920007782798207 PMC2758480

[28] Halladay J, Wong S, Jaffer S, Sinhababu A, Cyrus Khojasteh-Bakht S. Metabolic stability screen for drug discovery using cassette analysis and column switching. Drug Metab Lett. 2008;1(1):67–72. 10.2174/187231207779814364 19356021

[29] Di L, Kerns E, Ma X, Huang Y, Carter G. Applications of high throughput microsomal stability assay in drug discovery. Comb Chem High Throughput Screen. 2008;11(6):469–76. 18673274 10.2174/138620708784911429

[30] Knights KM, Stresser DM, Miners JO, Crespi CL. In vitro drug metabolism using liver microsomes. Curr Protoc Pharmacol. 2016(1). 10.1002/cpph.9. 10.1002/cpph.9 27636111

[31] Zhang L, Kasai S, Shono T. In vitro metabolism of pyriproxyfen by microsomes from susceptible and resistant housefly larvae. Arch Insect Biochem Physiol. 1998;37(3):215–24. 9465388 10.1002/(SICI)1520-6327(1998)37:3<215::AID-ARCH4>3.0.CO;2-R

[32] Suwanchaichinda C, Brattsten LB. Induction of microsomal cytochrome P450s by tire-leachate compounds, habitat components of *Aedes albopictus* mosquito larvae. Arch Insect Biochem Physiol. 2002;49(2):71–9. 11816022 10.1002/arch.10009

[33] Kasai S, Komagata O, Itokawa K, Shono T, Ng LC, Kobayashi M, et al. Mechanisms of pyrethroid resistance in the dengue mosquito vector, *Aedes aegypti*: target site insensitivity, penetration, and metabolism. PLoS Negl Trop Dis. 2014. 10.1371/journal.pntd.0002948. 24945250 10.1371/journal.pntd.0002948 PMC4063723

[34] David J, Ismail HM, Chandor-proust A, John M, Paine I, John M, et al. Role of cytochrome P450s in insecticide resistance: impact on the control of mosquito-borne diseases and use of insecticides on Earth. Philos Trans R Soc B. 2013;368:20120429. 10.1098/rstb.2012.0429 PMC3538419 23297352

[35] Pritchard MP, Ossetian R, Li DN, Henderson CJ, Burchell B, Wolf CR, et al. A general strategy for the expression of recombinant human cytochrome P450s in *Escherichia coli* using bacterial signal peptides: expression of CYP3A4, CYP2A6, and CYP2E1. Arch Biochem Biophys. 1997;345(2):342–54. 9308909 10.1006/abbi.1997.0265

[36] Lees RS, Ismail HM, Logan RAE, Malone D, Davies R, Anthousi A, et al. New insecticide screening platforms indicate that mitochondrial complex I inhibitors are susceptible to cross-resistance by mosquito P450s that metabolise pyrethroids. Sci Rep. 2020;10(1):1–10. Available from: 10.1038/s41598-020-73267-x. 10.1038/s41598-020-73267-x PMC7530702 33004954

[37] Riveron JM, Irving H, Ndula M, Barnes KG, Ibrahim SS, Paine MJI, et al. Directionally selected cytochrome P450 alleles are driving the spread of pyrethroid resistance in the major malaria vector *Anopheles funestus*. Proc Natl Acad Sci U S A. 2013;110(1):252–7. 23248325 10.1073/pnas.1216705110 PMC3538203

[38] Ibrahim SS, Riveron JM, Bibby J, Irving H, Yunta C, Paine MJI, et al. Allelic variation of cytochrome P450s drives resistance to Bednet insecticides in a major malaria vector. PLoS Genet. 2015;11(10):1–25. 10.1371/journal.pgen.1005618 PMC4627800 26517127

[39] Mugenzi LMJ, Tekoh A, Ibrahim TS, Muhammad S, Kouamo A, Wondji M, et al. MJ,. The duplicated P450s CYP6P9a/b drive carbamates and pyrethroids cross-resistance in the major African malaria vector *Anopheles funestus.* PLoS Genet. 2023;19(3):e1010678. Available from: 10.1371/journal.pgen.1010678. 10.1371/journal.pgen.1010678 PMC10089315 36972302

[40] Ibrahim SS, Kouamo MFM, Muhammad A, Irving H, Riveron JM, Tchouakui M, et al. Functional validation of endogenous redox partner cytochrome P450 reductase reveals the key P450s CYP6P9a / - b as broad substrate metabolizers conferring cross-resistance to different insecticide classes in *Anopheles funestus*. Int J Mol Sci. 2024;25:8092. 39125661 10.3390/ijms25158092 PMC11311542

[41] Hearn J, Djoko Tagne CS, Ibrahim SS, Tene-Fossog B, Mugenzi LMJ, Irving H, et al. Multi-omics analysis identifies a CYP9K1 haplotype conferring pyrethroid resistance in the malaria vector *Anopheles funestus* in East Africa. Mol Ecol. 2022;31(13):3642–57. 35546741 10.1111/mec.16497 PMC9321817

[42] Riveron JM, Ibrahim SS, Chanda E, Mzilahowa T, Cuamba N, Irving H, et al. The highly polymorphic CYP6M7 cytochrome P450 gene partners with the directionally selected CYP6P9a and CYP6P9b genes to expand the pyrethroid resistance front in the malaria vector *Anopheles funestus* in Africa. BMC Genomics. 2014;15(1):1–19. 25261072 10.1186/1471-2164-15-817 PMC4192331

[43] Ibrahim SS, Amvongo-Adjia N, Wondji MJ, Irving H, Riveron JM, Wondji CS. Pyrethroid resistance in the major malaria vector *Anopheles funestus* is exacerbated by overexpression and overactivity of the P450 CYP6AA1 across Africa. Genes (Basel). 2018;9(3):1–17. 10.3390/genes9030140 PMC5867861 29498712

[44] Stevenson BJ, Bibby J, Pignatelli P, Muangnoicharoen S, O’Neill PM, Lian LY, et al. Cytochrome P450 6m2 from the malaria vector *Anopheles gambiae* metabolizes pyrethroids: sequential metabolism of deltamethrin revealed. Insect Biochem Mol Biol. 2011;41(7):492–502. 10.1016/j.ibmb.2011.02.003. 21324359 10.1016/j.ibmb.2011.02.003

[45] Muller P, Warr E, Stevenson BJ, Pignatelli PM, Morgan JC, Yawson AE, et al. Field-caught permethrin-resistant *Anopheles gambiae* overexpress CYP6P3, a P450 that metabolises pyrethroids. PLoS Genet. 2008. 10.1371/journal.pgen.1000286. 19043575 10.1371/journal.pgen.1000286 PMC2583951

[46] Lucas ER, Nagi SC, Egyir-Yawson A, Essandoh J, Dadzie S, Chabi J, et al. Genome-wide association studies reveal novel loci associated with pyrethroid and organophosphate resistance in *Anopheles Gambiae* and *Anopheles coluzzii*. Nat Commun. 2023;14(1):1–23. 37587104 10.1038/s41467-023-40693-0 PMC10432508

[47] Irving H, Riveron JM, Ibrahim SS, Lobo NF, Wondji CS. Positional cloning of rp2 QTL associates the P450 genes CYP6Z1, CYP6Z3 and CYP6M7 with pyrethroid resistance in the malaria vector *Anopheles funestus*. Heredity (Edinb). 2012;109(6):383–92. 10.1038/hdy.2012.53. 22948188 10.1038/hdy.2012.53 PMC3499844

[48] Moyes CL, Lees RS, Yunta C, Walker KJ, Hemmings K, Oladepo F, et al. Assessing cross-resistance within the pyrethroids in terms of their interactions with key cytochrome P450 enzymes and resistance in vector populations. Parasit Vectors. 2021;14(1):1–13. Available from: 10.1186/s13071-021-04609-5. 10.1186/s13071-021-04609-5 PMC7893915 33602297

[49] McLaughlin LA, Niazi U, Bibby J, David JP, Vontas J, Hemingway J, et al. Characterization of inhibitors and substrates of *Anopheles Gambiae* CYP6Z2. Insect Mol Biol. 2008;17(2):125–35. 18353102 10.1111/j.1365-2583.2007.00788.x

[50] Vontas J, Grigoraki L, Morgan J, Tsakireli D, Fuseini G, Segura L, et al. Rapid selection of a pyrethroid metabolic enzyme CYP9K1 by operational malaria control activities. Proc Natl Acad Sci U S A. 2018;115(18):4619–24. 29674455 10.1073/pnas.1719663115 PMC5939083

[51] Nkya TE, Akhouayri I, Poupardin R, Batengana B, Mosha F, Magesa S, et al. Insecticide resistance mechanisms associated with different environments in the malaria vector *Anopheles gambiae*: a case study in Tanzania. Malar J. 2014. 10.1186/1475-2875-13-28. 24460952 10.1186/1475-2875-13-28 PMC3913622

[52] Weissenfels WD, Beyer M, Klein J, Rehm HJ. Microbial metabolism of fluoranthene: isolation and identification of ring fission products. Appl Microbiol Biotechnol. 1991;34:528–35. Available from: https://api.semanticscholar.org/CorpusID:22341915.

[53] Agbozu IE, Bayowa AV, Oghama OE. Critical analysis of polycyclic aromatic hydrocarbons ring size distribution in marshy soils and sediments in Warri City and its environs. Br. J. Appl. Sci. Technol 2017;20(6):1–16.

[54] Gilles M, Coetzee M. A supplement to anophelinae of Africa south of Sahara (Afro-tropical region). In Soth Africa. 1987. p. 55:1–143.

[55] Morgan JC, Irving H, Okedi LM, Steven A, Wondji CS. Pyrethroid resistance in an *Anopheles funestus* population from Uganda. PLoS ONE. 2010;5(7):1–8. 10.1371/journal.pone.0011872 PMC2912372 20686697

[56] Santolamazza F, Mancini E, Simard F, Qi Y, Tu Z, Della Torre A. Insertion polymorphisms of SINE200 retrotransposons within speciation Islands of *Anopheles Gambiae* molecular forms. Malar J. 2008;7:1–10. 18724871 10.1186/1475-2875-7-163 PMC2546427

[57] Harris C, Lambrechts L, Rousset F, Abate L, Nsango SE, Fontenille D, et al. Polymorphisms in *Anopheles Gambiae* immune genes associated with natural resistance to *Plasmodium falciparum*. PLoS Pathog. 2010. 10.1371/journal.ppat.1001112. 20862317 10.1371/journal.ppat.1001112 PMC2940751

[58] Lee SS, Scott JG. An improved method for preparation, stabilization, and storage of house fly (Diptera: Muscidae) microsomes. J Econ Entomol. 1989;82(6):1559–63. 2607028 10.1093/jee/82.6.1559

[59] Bradford M. A rapid and sensitive method for ther quantification of microgram quantities of protein utilizing the principle of protein-dye binding. Anal Biochem. 1976;(72):248–54. 10.1016/0003-2697(76)90527-3 942051

[60] Guengerich FP, Martin MV, Sohl CD, Cheng Q. Measurement of cytochrome P450 and NADPH-cytochrome P450 reductase. Nat Protoc. 2009;4(9):1245–51. 19661994 10.1038/nprot.2009.121 PMC3843963

[61] Sato RYO, Omura T. The carbon monoxide-binding pigment of liver microsomes. J Biol Chem. 1964;239(7):2370–8. 10.1016/S0021-9258(20)82244-3. 14209971

[62] Lakens D. Calculating and reporting effect sizes to facilitate cumulative science: a practical primer for t-tests and ANOVAs. Front Psychol. 2013;4(NOV):1–12. 24324449 10.3389/fpsyg.2013.00863 PMC3840331

[63] Nkya TE, Akhouayri I, Kisinza W, David JP. Impact of environment on mosquito response to pyrethroid insecticides: facts, evidences and prospects. Insect Biochem Mol Biol. 2013;43(4):407–16. 10.1016/j.ibmb.2012.10.006. 23123179 10.1016/j.ibmb.2012.10.006

[64] Kamdem C, Fouet C, Gamez S, White BJ. Pollutants and insecticides drive local adaptation in African malaria mosquitoes. Mol Biol Evol. 2017;34(5):1261–75. 28204524 10.1093/molbev/msx087 PMC5400387

[65] Awolola TS, Oduola AO, Obansa JB, Chukwurar NJ, Unyimadu JP. *Anopheles gambiae s.s.* breeding in polluted water bodies in urban Lagos, southwestern Nigeria. J Vector Borne Dis. 2007;44(December):241–4. 18092529

[66] Sterkel M, Haines LR, Casas-Sánchez A, Adung’a VO, Vionette-Amaral RJ, Quek S, et al. Repurposing the orphan drug Nitisinone to control the transmission of African trypanosomiasis. PLoS Biol. 2021. 10.1371/journal.pbio.3000796. 33497373 10.1371/journal.pbio.3000796 PMC7837477

[67] Wilkinson CF, Brattsten LB. Microsomal drug metabolizing enzymes in insects. Drug Metab Rev. 1972;1(1):153–227.

[68] Nolden M, Brockmann A, Ebbinghaus-Kintscher U, Brueggen K-U, Horstmann S, Paine MJI, et al. Towards understanding transfluthrin efficacy in a pyrethroid-resistant strain of the malaria vector *Anopheles funestus* with special reference to cytochrome P450-mediated detoxification. Current Research in Parasitology & Vector-Borne Diseases. 2021;1:100041. 10.1016/j.crpvbd.2021.100041. 35284893 10.1016/j.crpvbd.2021.100041 PMC8906121

[69] Brattsten LB, Gunderson CA. Isolation of insect microsomal oxidases by rapid centrifugation. Pestic Biochem Physiol. 1981;16(3):187–98.

[70] Srdič M, Fessner ND, Yildiz D, Glieder A, Spiertz M, Schwaneberg U. Preparative production of functionalized (N-and O-heterocyclic) polycyclic aromatic hydrocarbons by human cytochrome P450 3A4 in a bioreactor. Biomolecules. 2022. 10.3390/biom12020153. 35204652 10.3390/biom12020153 PMC8961652

[71] Shimada T, Murayama N, Kakimoto K, Takenaka S, Lim YR, Yeom S, et al. Oxidation of 1-chloropyrene by human CYP1 family and CYP2A subfamily cytochrome P450 enzymes: catalytic roles of two CYP1B1 and five CYP2A13 allelic variants. Xenobiotica. 2018;48(6):565–75. 10.1080/00498254.2017.1347306. 28648140 10.1080/00498254.2017.1347306 PMC5780263

[72] Feyreisen R, Durst F. Ecdysterone biosynthesis: a microsomal Cytochrome-P‐450‐linked ecdysone 20‐monooxygenase from tissues of the African migratory locust. Eur J Biochem. 1978;88(1):37–47. 27363 10.1111/j.1432-1033.1978.tb12420.x

[73] Iyanagi T. Molecular mechanism of phase I and phase II Drug-Metabolizing enzymes: implications for detoxification. Int Rev Cytol. 2007;260:35–112. 17482904 10.1016/S0074-7696(06)60002-8

[74] Adolfi A, Poulton B, Anthousi A, Macilwee S, Ranson H, Lycett GJ. Functional genetic validation of key genes conferring insecticide resistance in the major African malaria vector, *Anopheles gambiae*. Proc Natl Acad Sci U S A. 2019;116(51):25764–72. 31801878 10.1073/pnas.1914633116 PMC6926047

[75] Edi CV, Djogbénou L, Jenkins AM, Regna K, Muskavitch MAT, Poupardin R, et al. CYP6 P450 enzymes and ACE-1 duplication produce extreme and multiple insecticide resistance in the malaria mosquito *Anopheles Gambiae*. PLoS Genet. 2014. 10.1371/journal.pgen.1004236. 24651294 10.1371/journal.pgen.1004236 PMC3961184

[76] Chandor-Proust A, Bibby J, Régent-Kloeckner M, Roux J, Guittard-Crilat E, Poupardin R, et al. The central role of mosquito cytochrome P450 CYP6Zs in insecticide detoxification revealed by functional expression and structural mode. Biochem J. 2013;455(1):75–85. 23844938 10.1042/BJ20130577 PMC3778711

[77] Nikou D, Ranson H, Hemingway J. An adult-specific CYP6 P450 gene is overexpressed in a pyrethroid- resistant strain of the malaria vector. Anopheles Gambiae Gene. 2003;318(1– 14585502 10.1016/s0378-1119(03)00763-7

[78] Vontas J, Hemingway J, Ranson H, Sutcliffe MJ, Paine MJI. Characterization of inhibitors and substrates of Anopheles Gambiae CYP6Z2. Insect Mol Biol. 2008;17(February):125–35. 18353102 10.1111/j.1365-2583.2007.00788.x

